# Supplementary material for: Pyruvate Dehydrogenase Kinase 1 inhibition mediated oxidative phosphorylation enhancement in cartilage promotes osteoarthritis progression
Source: BMC Musculoskelet Disord. 2023 Jul 20;24:597. doi: 10.1186/s12891-023-06585-6 (PMC10357736; doi:10.1186/s12891-023-06585-6)
Supplement: Supplementary file 1 — Additional file 1. [file 12891_2023_6585_MOESM1_ESM.zip › BP.Gsea.1653623667859/GOBP_HISTONE_DEACETYLATION.html]

Details for gene set GOBP\_HISTONE\_DEACETYLATION[GSEA]

|  || Dataset | 55457\_collapsed\_to\_symbols.cls.cls #OA\_versus\_normal.cls.cls #OA\_versus\_normal\_repos |
| Phenotype | cls.cls#OA\_versus\_normal\_repos |
| Upregulated in class | normal |
| GeneSet | GOBP\_HISTONE\_DEACETYLATION |
| Enrichment Score (ES) | -0.44322917 |
| Normalized Enrichment Score (NES) | -1.8135349 |
| Nominal p-value | 0.0 |
| FDR q-value | 0.08814962 |
| FWER p-Value | 0.967 |
Table: GSEA Results Summary

  

Fig 1: Enrichment plot: GOBP\_HISTONE\_DEACETYLATION      
 Profile of the Running ES Score & Positions of GeneSet Members on the Rank Ordered List

  

| SYMBOL | TITLE | RANK IN GENE LIST | RANK METRIC SCORE | RUNNING ES | CORE ENRICHMENT || 1 | TBL1XR1 | TBL1X receptor 1 [Source:HGNC Symbol;Acc:HGNC:29529] | 1070 | 0.501 | -0.0635 | No |
| 2 | RBBP4 | "RB binding protein 4, chromatin remodeling factor [Source:HGNC Symbol;Acc:HGNC:9887]" | 1181 | 0.480 | -0.0530 | No |
| 3 | TADA3 | transcriptional adaptor 3 [Source:HGNC Symbol;Acc:HGNC:19422] | 1295 | 0.459 | -0.0436 | No |
| 4 | RRP8 | ribosomal RNA processing 8 [Source:HGNC Symbol;Acc:HGNC:29030] | 1673 | 0.402 | -0.0570 | No |
| 5 | MBD2 | methyl-CpG binding domain protein 2 [Source:HGNC Symbol;Acc:HGNC:6917] | 1958 | 0.366 | -0.0646 | No |
| 6 | PINK1 | PTEN induced kinase 1 [Source:HGNC Symbol;Acc:HGNC:14581] | 2097 | 0.350 | -0.0615 | No |
| 7 | SPHK2 | sphingosine kinase 2 [Source:HGNC Symbol;Acc:HGNC:18859] | 2478 | 0.305 | -0.0790 | No |
| 8 | MBIP | MAP3K12 binding inhibitory protein 1 [Source:HGNC Symbol;Acc:HGNC:20427] | 2626 | 0.287 | -0.0791 | No |
| 9 | RBBP7 | "RB binding protein 7, chromatin remodeling factor [Source:HGNC Symbol;Acc:HGNC:9890]" | 2777 | 0.272 | -0.0799 | No |
| 10 | MORF4L1 | mortality factor 4 like 1 [Source:HGNC Symbol;Acc:HGNC:16989] | 3984 | 0.157 | -0.1677 | No |
| 11 | SIRT3 | sirtuin 3 [Source:HGNC Symbol;Acc:HGNC:14931] | 4009 | 0.155 | -0.1634 | No |
| 12 | PRKD1 | protein kinase D1 [Source:HGNC Symbol;Acc:HGNC:9407] | 4123 | 0.144 | -0.1665 | No |
| 13 | HDAC5 | histone deacetylase 5 [Source:HGNC Symbol;Acc:HGNC:14068] | 4336 | 0.125 | -0.1781 | No |
| 14 | SREBF1 | sterol regulatory element binding transcription factor 1 [Source:HGNC Symbol;Acc:HGNC:11289] | 4729 | 0.093 | -0.2050 | No |
| 15 | HOPX | HOP homeobox [Source:HGNC Symbol;Acc:HGNC:24961] | 4803 | 0.087 | -0.2072 | No |
| 16 | PHB | prohibitin [Source:HGNC Symbol;Acc:HGNC:8912] | 5102 | 0.064 | -0.2279 | No |
| 17 | HDAC9 | histone deacetylase 9 [Source:HGNC Symbol;Acc:HGNC:14065] | 5183 | 0.058 | -0.2318 | No |
| 18 | TP53 | tumor protein p53 [Source:HGNC Symbol;Acc:HGNC:11998] | 5511 | 0.032 | -0.2561 | No |
| 19 | MSL3 | MSL complex subunit 3 [Source:HGNC Symbol;Acc:HGNC:7370] | 5596 | 0.024 | -0.2617 | No |
| 20 | NCAPG2 | non-SMC condensin II complex subunit G2 [Source:HGNC Symbol;Acc:HGNC:21904] | 5700 | 0.016 | -0.2691 | No |
| 21 | MBD3 | methyl-CpG binding domain protein 3 [Source:HGNC Symbol;Acc:HGNC:6918] | 5795 | 0.008 | -0.2761 | No |
| 22 | RCOR3 | REST corepressor 3 [Source:HGNC Symbol;Acc:HGNC:25594] | 6014 | -0.006 | -0.2928 | No |
| 23 | SUV39H1 | suppressor of variegation 3-9 homolog 1 [Source:HGNC Symbol;Acc:HGNC:11479] | 6028 | -0.007 | -0.2936 | No |
| 24 | ING2 | inhibitor of growth family member 2 [Source:HGNC Symbol;Acc:HGNC:6063] | 6150 | -0.016 | -0.3024 | No |
| 25 | SIRT2 | sirtuin 2 [Source:HGNC Symbol;Acc:HGNC:10886] | 6279 | -0.026 | -0.3114 | No |
| 26 | DR1 | down-regulator of transcription 1 [Source:HGNC Symbol;Acc:HGNC:3017] | 6369 | -0.033 | -0.3170 | No |
| 27 | BRMS1 | BRMS1 transcriptional repressor and anoikis regulator [Source:HGNC Symbol;Acc:HGNC:17262] | 6602 | -0.050 | -0.3331 | No |
| 28 | PRKD2 | protein kinase D2 [Source:HGNC Symbol;Acc:HGNC:17293] | 7011 | -0.081 | -0.3617 | No |
| 29 | FOXP3 | forkhead box P3 [Source:HGNC Symbol;Acc:HGNC:6106] | 7035 | -0.082 | -0.3602 | No |
| 30 | SKI | SKI proto-oncogene [Source:HGNC Symbol;Acc:HGNC:10896] | 7349 | -0.106 | -0.3804 | No |
| 31 | SGF29 | SAGA complex associated factor 29 [Source:HGNC Symbol;Acc:HGNC:25156] | 7363 | -0.107 | -0.3772 | No |
| 32 | PER2 | period circadian regulator 2 [Source:HGNC Symbol;Acc:HGNC:8846] | 7663 | -0.132 | -0.3953 | No |
| 33 | SAP30BP | SAP30 binding protein [Source:HGNC Symbol;Acc:HGNC:30785] | 7739 | -0.137 | -0.3957 | No |
| 34 | HDAC6 | histone deacetylase 6 [Source:HGNC Symbol;Acc:HGNC:14064] | 7833 | -0.143 | -0.3972 | No |
| 35 | HDAC1 | histone deacetylase 1 [Source:HGNC Symbol;Acc:HGNC:4852] | 7911 | -0.148 | -0.3974 | No |
| 36 | APLNR | apelin receptor [Source:HGNC Symbol;Acc:HGNC:339] | 8001 | -0.155 | -0.3981 | No |
| 37 | HDAC4 | histone deacetylase 4 [Source:HGNC Symbol;Acc:HGNC:14063] | 8416 | -0.187 | -0.4230 | Yes |
| 38 | PRDM5 | PR/SET domain 5 [Source:HGNC Symbol;Acc:HGNC:9349] | 8486 | -0.193 | -0.4207 | Yes |
| 39 | SIRT6 | sirtuin 6 [Source:HGNC Symbol;Acc:HGNC:14934] | 8532 | -0.196 | -0.4165 | Yes |
| 40 | AKAP8 | A-kinase anchoring protein 8 [Source:HGNC Symbol;Acc:HGNC:378] | 8659 | -0.205 | -0.4181 | Yes |
| 41 | ZBTB7B | zinc finger and BTB domain containing 7B [Source:HGNC Symbol;Acc:HGNC:18668] | 8702 | -0.209 | -0.4131 | Yes |
| 42 | SIRT7 | sirtuin 7 [Source:HGNC Symbol;Acc:HGNC:14935] | 8733 | -0.211 | -0.4070 | Yes |
| 43 | CHD4 | chromodomain helicase DNA binding protein 4 [Source:HGNC Symbol;Acc:HGNC:1919] | 8917 | -0.226 | -0.4124 | Yes |
| 44 | SIRT1 | sirtuin 1 [Source:HGNC Symbol;Acc:HGNC:14929] | 8947 | -0.227 | -0.4056 | Yes |
| 45 | MAPK8 | mitogen-activated protein kinase 8 [Source:HGNC Symbol;Acc:HGNC:6881] | 8956 | -0.228 | -0.3971 | Yes |
| 46 | ZZZ3 | zinc finger ZZ-type containing 3 [Source:HGNC Symbol;Acc:HGNC:24523] | 8991 | -0.231 | -0.3906 | Yes |
| 47 | RCOR1 | REST corepressor 1 [Source:HGNC Symbol;Acc:HGNC:17441] | 9120 | -0.241 | -0.3910 | Yes |
| 48 | HDAC2 | histone deacetylase 2 [Source:HGNC Symbol;Acc:HGNC:4853] | 9359 | -0.260 | -0.3993 | Yes |
| 49 | TBL1Y | transducin beta like 1 Y-linked [Source:HGNC Symbol;Acc:HGNC:18502] | 9416 | -0.266 | -0.3931 | Yes |
| 50 | RBM14 | RNA binding motif protein 14 [Source:HGNC Symbol;Acc:HGNC:14219] | 9663 | -0.288 | -0.4008 | Yes |
| 51 | SFPQ | splicing factor proline and glutamine rich [Source:HGNC Symbol;Acc:HGNC:10774] | 9747 | -0.296 | -0.3955 | Yes |
| 52 | KAT2A | lysine acetyltransferase 2A [Source:HGNC Symbol;Acc:HGNC:4201] | 9799 | -0.301 | -0.3875 | Yes |
| 53 | CTBP1 | C-terminal binding protein 1 [Source:HGNC Symbol;Acc:HGNC:2494] | 10188 | -0.339 | -0.4043 | Yes |
| 54 | CHD3 | chromodomain helicase DNA binding protein 3 [Source:HGNC Symbol;Acc:HGNC:1918] | 10269 | -0.347 | -0.3967 | Yes |
| 55 | MTA2 | metastasis associated 1 family member 2 [Source:HGNC Symbol;Acc:HGNC:7411] | 10600 | -0.383 | -0.4072 | Yes |
| 56 | ELK4 | ETS transcription factor ELK4 [Source:HGNC Symbol;Acc:HGNC:3326] | 10862 | -0.411 | -0.4113 | Yes |
| 57 | KDM5A | lysine demethylase 5A [Source:HGNC Symbol;Acc:HGNC:9886] | 11097 | -0.443 | -0.4119 | Yes |
| 58 | TBL1X | transducin beta like 1 X-linked [Source:HGNC Symbol;Acc:HGNC:11585] | 11263 | -0.466 | -0.4063 | Yes |
| 59 | MIER2 | MIER family member 2 [Source:HGNC Symbol;Acc:HGNC:29210] | 11375 | -0.483 | -0.3957 | Yes |
| 60 | GATAD2A | GATA zinc finger domain containing 2A [Source:HGNC Symbol;Acc:HGNC:29989] | 11985 | -0.590 | -0.4198 | Yes |
| 61 | MORF4L2 | mortality factor 4 like 2 [Source:HGNC Symbol;Acc:HGNC:16849] | 12061 | -0.610 | -0.4014 | Yes |
| 62 | YEATS2 | YEATS domain containing 2 [Source:HGNC Symbol;Acc:HGNC:25489] | 12179 | -0.639 | -0.3851 | Yes |
| 63 | TADA2A | transcriptional adaptor 2A [Source:HGNC Symbol;Acc:HGNC:11531] | 12217 | -0.650 | -0.3622 | Yes |
| 64 | REST | RE1 silencing transcription factor [Source:HGNC Symbol;Acc:HGNC:9966] | 12382 | -0.703 | -0.3471 | Yes |
| 65 | NIPBL | NIPBL cohesin loading factor [Source:HGNC Symbol;Acc:HGNC:28862] | 12385 | -0.703 | -0.3193 | Yes |
| 66 | PER1 | period circadian regulator 1 [Source:HGNC Symbol;Acc:HGNC:8845] | 12496 | -0.746 | -0.2982 | Yes |
| 67 | UCN | urocortin [Source:HGNC Symbol;Acc:HGNC:12516] | 12536 | -0.765 | -0.2709 | Yes |
| 68 | HDAC11 | histone deacetylase 11 [Source:HGNC Symbol;Acc:HGNC:19086] | 12601 | -0.799 | -0.2442 | Yes |
| 69 | VEGFA | vascular endothelial growth factor A [Source:HGNC Symbol;Acc:HGNC:12680] | 12629 | -0.813 | -0.2140 | Yes |
| 70 | AKAP8L | A-kinase anchoring protein 8 like [Source:HGNC Symbol;Acc:HGNC:29857] | 12665 | -0.836 | -0.1835 | Yes |
| 71 | BCL6 | BCL6 transcription repressor [Source:HGNC Symbol;Acc:HGNC:1001] | 12667 | -0.838 | -0.1503 | Yes |
| 72 | MTA1 | metastasis associated 1 [Source:HGNC Symbol;Acc:HGNC:7410] | 12792 | -0.952 | -0.1221 | Yes |
| 73 | SIN3B | SIN3 transcription regulator family member B [Source:HGNC Symbol;Acc:HGNC:19354] | 12809 | -0.984 | -0.0843 | Yes |
| 74 | HDAC7 | histone deacetylase 7 [Source:HGNC Symbol;Acc:HGNC:14067] | 12870 | -1.131 | -0.0441 | Yes |
| 75 | BAZ2A | bromodomain adjacent to zinc finger domain 2A [Source:HGNC Symbol;Acc:HGNC:962] | 12879 | -1.166 | 0.0016 | Yes |
Table: GSEA details [plain text format]

  

Fig 2: GOBP\_HISTONE\_DEACETYLATION      
 Blue-Pink O' Gram in the Space of the Analyzed GeneSet

  

Fig 3: GOBP\_HISTONE\_DEACETYLATION: Random ES distribution      
 Gene set null distribution of ES for **GOBP\_HISTONE\_DEACETYLATION**
